# Editorial: Hockey: testing and performance

**DOI:** 10.3389/fspor.2024.1364470

**Published:** 2024-01-25

**Authors:** Franck Brocherie, Bernd J. Stetter, Dmitri Poltavski, Jeppe F. Vigh-Larsen

**Affiliations:** ^1^ Laboratory Sport, Expertise and Performance (EA 7370), French Institute of Sport, Paris, France; ^2^ Institute of Sports and Sports Science, Karlsruhe Institute of Technology (KIT), Karlsruhe, Germany; ^3^ Department of Psychology, University of North Dakota, Grand Forks, ND, United States; ^4^ Department of Sports Science and Clinical Biomechanics, University of Southern Denmark, Odense, Denmark

**Keywords:** field hockey, ice hockey, developmental pathways, metabolic systems, mechanical capabilities, skating, priming, informed decision

**Editorial on the Research Topic** Hockey: testing and performance

## The research topic

Hockey is a term used in reference to either the summer sport of field hockey (introduced at the Olympic Games in 1908) or the winter ice hockey (introduced at the Olympic Games in 1920). It also encompasses related variant games such as bandy, inline/roller hockey, and floor hockey that are played outdoor or indoor on different surfaces (ice, polypropylene, polyvinyl chloride (PVC), or wooden surfaces) while holding a stick. Most of these disciplines appear underrepresented in the different fields (e.g., conditioning, physiology and nutrition, biomechanics, coaching, psychology) of the sports sciences and medicine literature (1, 2). Therefore, as part of a Frontiers in Sports and Active Living initiative to launch a series of Research Topics designated to increase the scientific output in “underrepresented” sports, we called on novel hockey sports-related scientific contributions addressing the determinants of performance (including technical, physiological, biomechanical, psychological, and nutritional factors), training and testing, development pathways, injury prevention, and rehabilitation among others at the amateur and elite levels.

## The inputs

A total of three original researches, one brief research report, one systematic review, and one opinion, are included in this Research Topic. Except a single contribution in field hockey, all the others were related to ice hockey with a strong representation from North America (USA and Canada). These contributions ranged from ice hockey-specific developmental issues (Garland et al.) and off-ice testing (Glaude-Roy et al., Bournival et al.) to team-sport generalizable concepts such as priming (Brocherie and Perez), offensive–defensive team dynamics (Mizawa et al.), and psychological impact of blowouts on subsequent team performance (Chachad et al.).

Ice hockey is among the sports where the belief in early specialization is widespread, notably via the contentious 10-year (or 10,000 h rule) model (3) to demonstrate expertise in skating. Despite several recommendations (e.g., International Olympic Committee) to delay sport specialization among children (4), Garland et al. reported that young ice hockey players were involved in competition prior to the age suggested by Hockey Canada and the *Developmental Model of Sport Participation* (5). Involvement in recreational competition started at the age of 5 and most players at the age of 7 were training for 8 or more months per year. Interestingly, it has previously been shown that those who participate in multiple sports at the age of 12 show better physical performance (6). These results are important to consider in the future developmental pathway to avoid premature peak level of play (and its psychosocial consequences) and/or increased risk of injury.

Another specificity of ice hockey performance relates to its physiological/physical assessment, based on off- and on-ice tests and their respective pros and cons. For instance, whether forward skating sprint mechanical determinants were inferred from off- and on-ice force–velocity profiles was recently explored (7). Glaude-Roy et al. focused on the off-ice association of over-ground sprinting force–velocity capabilities with anaerobic capacities (i.e., Wingate and repeated-sprint testing) in highly competitive adolescent (15–17 years old) male and female ice hockey players. While pooled data relate force capability with anaerobic capacities, gender-dependent variation was highlighted. However, the critical question of transferability of off-ice fitness to on-ice performance remains a key concern, calling for future investigation. In this view, Bournival et al. proposed a compilation of on-ice tests used over the last 20 years among different age groups, genders, and playing levels. The physiological and physical ice hockey-specific attributes assessed in an ecological setting (i.e., on the ice) were skating acceleration and speed, change of direction and agility, repeated-sprint ability, and aerobic capacity. Ice hockey practitioners can benefit from this inventory by selecting on-ice tests adapted to the characteristics of their players.

The following three contributions seem more generalizable, with concepts applicable in other training/game situations or team-sport settings: First, the empirical use of morning skate on game day, also implemented in other sports as “activation” or “muscle wake-up” has been challenged by Brocherie and Perez. Besides disputable scientific evidences (e.g., psychological behavior, chronobiology, potentiation or priming effect), the decision to conduct or cancel a mandatory or optional morning skate on game day, or possible alternatives (e.g., off-ice priming session, tactical video analysis, recovery, and/or medical care) is team-, context-, and player-dependent. To help practitioners involved in ice hockey, a decision tree has been proposed, with possible usefulness in other sport-specific settings.

Second, the pass-chaining situation can be illustrative of the fluctuation of the attack–defense collective behavior and its effects on game momentum in team sports (8). Mizawa et al. provide insights regarding the spatiotemporal structure of the collective pass-chaining action. For that, they quantified the “defensive pressure distribution” (i.e., the functional distance of defenders to reach their opponents’ ball) on the pass trajectory during three-on-three small-sided games in expert vs. intermediate Japanese collegiate field hockey players. With higher expertise, the attack–defense equilibrium is stabilized with superior shooting opportunities, mainly due to a short time constant (i.e., < 1 s) for passing actions. This may have some practical implications in small-sided games adjustments (e.g., playing surface, number of players) to enhance attack–defense collective behavior.

Third, Chachad et al. examined the potential carryover effect of blowouts (i.e., a victory or a loss with a large margin) on subsequent game performance in the National Hockey League. After adjustment for location of the subsequent game, number of time zones from the home base city, whether the subsequent game was a back-to-back game, and winning percentages of the team and opponent, no significant over- or underperformance were found for teams benefiting or suffering from a blowout.

## Closing observations

This Research Topic envisioned boosting scientific visibility of “underrepresented” hockey sports in the sports sciences and medicine literature. Surprisingly, except for the two major hockey sports—i.e., field and ice hockey—no manuscript considering bandy, inline/roller hockey, or floor hockey was submitted. It is clearly evident that a never-ending effort is needed to address relevant research questions related to field and ice hockey, as well as their related variants, some of which are developing rapidly, along with a growing interest from players, coaches, and equipment manufacturers. As illustrated here, it is worth mentioning that some content may be relevant to other sports practitioners.
